# Collection of Data on Sex, Sexual Orientation, and Gender Identity by U.S. Public Health Data and Monitoring Systems, 2015–2018

**DOI:** 10.3390/ijerph182212189

**Published:** 2021-11-20

**Authors:** Alissa C. Kress, Asia Asberry, Julio Dicent Taillepierre, Michelle M. Johns, Pattie Tucker, Ana Penman-Aguilar

**Affiliations:** 1 Office of Women’s Health, Centers for Disease Control and Prevention, Atlanta, GA 30341, USA; pjt1@cdc.gov; 2 Department of Health Promotion and Behavior, University of Georgia College of Public Health, Athens, GA 30602, USA; asia.asberry143@gmail.com; 3 Office of Minority Health and Health Equity, Centers for Disease Control and Prevention, Atlanta, GA 30341, USA; jtt5@cdc.gov (J.D.T.); bpv4@cdc.gov (A.P.-A.); 4 Centers for Disease Control and Prevention, Division of Adolescent and School Health, Atlanta, GA 30329, USA; yrm9@cdc.gov

**Keywords:** women’s health, sexual and gender minorities, data collection

## Abstract

We aimed to assess Centers for Disease Control and Prevention (CDC) data systems on the extent of data collection on sex, sexual orientation, and gender identity as well as on age and race/ethnicity. Between March and September 2019, we searched 11 federal websites to identify CDC-supported or -led U.S. data systems active between 2015 and 2018. We searched the systems’ website, documentation, and publications for evidence of data collection on sex, sexual orientation, gender identity, age, and race/ethnicity. We categorized each system by type (disease notification, periodic prevalence survey, registry/vital record, or multiple sources). We provide descriptive statistics of characteristics of the identified systems. Most (94.1%) systems we assessed collected data on sex. All systems collected data on age, and approximately 80% collected data on race/ethnicity. Only 17.7% collected data on sexual orientation and 5.9% on gender identity. Periodic prevalence surveys were the most common system type for collecting all the variables we assessed. While most U.S. public health data and monitoring systems collect data disaggregated by sex, age, and race/ethnicity, far fewer do so for sexual orientation or gender identity. Standards and examples exist to aid efforts to collect and report these vitally important data. Additionally important is increasing accessibility and appropriately tailored dissemination of reports of these data to public health professionals and other collaborators.

## 1. Introduction

Public health data and monitoring systems and tools collect, analyze, disseminate, and evaluate public health data from a variety of sources to address a range of public health needs [1,2]. As a national leader in public health surveillance, the Centers for Disease Control and Prevention (CDC) plays an important role in developing public health data systems as well as aggregating, analyzing, and disseminating public health data [3]. A previous report reviewed CDC-sponsored data systems and found they would benefit from more complete and accurate data collection on race, ethnicity, language, and nativity [4]. To our knowledge, no report has yet described data collection of sex- and gender-related variables across data systems at CDC. Therefore, in consideration of the leadership role of CDC, the aim of our study was to assess the extent of data collection by CDC data and monitoring systems on sex, sexual orientation, and gender identity, as well as age and race/ethnicity.

Despite making up approximately half of the population, women have historically been underrepresented in health research [5]. Over the last four decades, however, there has been an increased focus on women’s health issues [5], and since the early 2000s, there has been a renewed interest for the inclusion of women in research studies as well as publication of sex- and gender-focused analyses [5,6,7,8,9,10]. Sex refers to biological sex assigned at birth (e.g., male, female), while gender refers to the social or cultural norms and roles expected of individuals based on their sex (e.g., what social or behavioral dimensions make a woman or man) [11]. Conducting and reporting sex- and gender-stratified analyses can help improve our understanding not only of women’s health, but men’s health as well [6,7,8]. For example, in 2017, the top two leading causes of death—heart disease and cancer—were the same for men and women [12]. However, women and men experience heart diseases and cancers differently; women having a heart attack are more likely to have atypical symptoms, such as unusual fatigue, and women have a higher risk of developing right-sided (proximal) colon cancer, which is often at a more advanced stage at diagnosis [13,14].

To an even greater extent, health research that takes into consideration sexual orientation and gender identity (SOGI) is limited [15]. Sexual orientation is a multi-dimensional construct consisting of sexual attraction, sexual behavior, and sexual identity, the most common descriptors of which are lesbian (L), gay (G), bisexual (B), and heterosexual [11]. Gender identity refers to an internal sense of being male, female, a combination, or neither; the term transgender (T) refers to individuals whose gender identity differs from their sex at birth, while cisgender refers to individuals whose gender identity aligns with their sex at birth [11]. Despite the dearth of research on these populations, disparities are known to exist between LGBT and heterosexual/cisgender individuals. For example, gay/lesbian women are more likely than heterosexual women to currently smoke cigarettes and to have ever used e-cigarettes [16], and women who have sex with women are less likely than those who have sex with men to access preventive health services, such as Pap tests and mammograms [17,18]. LGBT individuals are also at increased risk for depression and suicide, substance use, and are more likely to report experiencing stigma, discrimination, and violence than their heterosexual and cisgender counterparts [15,19,20,21,22,23].

While disaggregation of data by sex and SOGI is a good starting point [7,9], intersectionality with related social, individual, and health care factors as well as with the influence of gender norms also requires consideration [5,7]. The recently developed Sex and Gender Equity in Research (SAGER) guidelines encourage reporting of data not only by sex but also other relevant characteristics (e.g., socioeconomic status) from a sex and gender perspective [8]. Similarly, just as there are health differences between sexes, there are important differences within the sexes. For example, women and men of various racial and ethnic groups (e.g., Asians) and more detailed subgroups (e.g., persons of Chinese origin) may have differing health profiles or needs, as may women (and men) who are veterans and non-veterans or who have or do not have a disability, among others [5]. Furthermore, SOGI subgroups have not been sufficiently researched; for example, a recent study found that past-year suicide attempts were higher for bisexual women than gay/lesbian women for certain age and race/ethnicity groups [15,21]. Therefore, it is important not only to collect data on sex, sexual orientation, and gender identity but also on other relevant socio-demographic characteristics, such as age and race/ethnicity.

## 2. Materials and Methods

Between March and September 2019, we searched 11 CDC and other federal websites [24,25,26,27,28,29,30,31,32,33,34] to identify data systems that met the following inclusion criteria: (1) CDC supported or led, (2) active between the years 2015 and 2018, (3) recorded data on an ongoing or periodic basis (i.e., more than once), (4) did not rely on preexisting data sources, (5) monitored human health, and (6) monitored data only in the U.S. For each system identified meeting all six of these criteria, we searched the system’s publicly available website, documentation, and publications for evidence of data collection on sex-related variables: sex (e.g., male, female), sexual orientation (e.g., heterosexual, gay, lesbian, etc.), and gender identity (e.g., cisgender, transgender, nonbinary, etc.). A data system was considered to collect data on sex-related variables if we were able to identify at least one instance of the system having done so. Additionally, we assessed whether each system collected data on age (either a specific age or age range) and race/ethnicity (using some version of the 1997 OMB federally required minimum data standards five racial categories (American Indian or Alaska Native, Asian, Black or African American, Native Hawaiian or other Pacific Islander, and White) and two ethnicity categories (Hispanic or Latino and not Hispanic or Latino)). If we were unable to find this information on the system’s website, we attempted to contact the system owner or point of contact.

We categorized each system by type as disease notification, periodic prevalence survey, registry/vital record, or multiple sources (Table 1); we based these categories on those we identified from the literature [35,36]. If a system fit into more than one category (e.g., if a system displayed registry and survey data), we assigned it to the multiple sources category.

Data captured through our assessment of data systems included: information on meeting inclusion criteria (yes or no to each criteria); evidence of data collection on sex, SOGI, age, and race/ethnicity (yes or no); type of system; URL for accessing the system’s data, documentation, and/or data reports; point of contact for the system (if available); and any general notes about the system. Identification and assessment of data systems was completed independently by two analysts, and any discrepancies noted were jointly discussed and reconciled. We entered data collected from our assessment into Excel, and then, after any discrepancies were reconciled, we imported the data into SAS version 9.4 for analysis. In this report, we provide descriptive statistics of characteristics of the identified systems overall and by type of system. Among those systems that collected data on sex or SOGI, we also provide descriptive statistics on those collecting data on age and race/ethnicity.

## 3. Results

We identified a total of 154 data systems, 51 of which met our inclusion criteria. The systems included in our analysis represented a variety of topic areas, such as chronic and acute diseases, mortality, injury, health-related behaviors, and hospital-related topics. See Table 1 for an example of a data system in each category.

Of the 51 included systems, three were female-specific (none were male-specific), while 48 encompassed both sexes. Female-specific systems were considered to have collected data on sex. Overall, nearly all systems collected (94.1%) data on sex. Fewer systems collected data on sexual orientation (17.7%) and gender identity (5.9%); all data systems that captured data on sexual orientation and/or gender identity also collected data on sex. All systems we assessed collected data on age, and about 4 in 5 (78.4%) collected data on race/ethnicity. The most common types of systems included were disease notifications (52.9%), followed by periodic prevalence surveys (33.3%) and registries/vital records (11.8%). The three female-specific data systems were all related to reproductive health and/or pregnancy; none of them collected data on sexual orientation or gender identity, and all of them collected data on age and race/ethnicity. Of these systems, two were disease notifications, and one was a periodic prevalence survey (Table 2).

**Figure 1:**
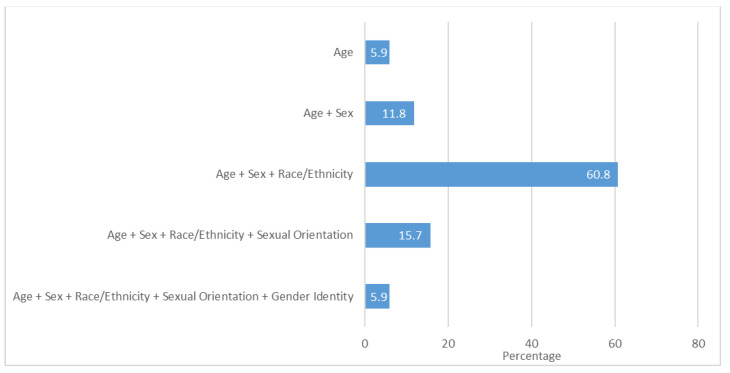
Percentage of U.S. public health data and monitoring systems collecting data on sex, sexual orientation, gender identity, age, and race/ethnicity, 2015–2018.

Approximately 1 in 5 systems (21.6%) collected data on age, sex, race/ethnicity, and sexual orientation (15.7%) or all of those variables plus gender identity (5.9%). Most systems collected data only on age, sex, and race/ethnicity (60.8%). Fewer systems collected data on age only (5.9%), or age and sex only (11.8%) (Figure 1).

All periodic prevalence surveys and registries/vital records collected data on sex, as did the one system based on multiple sources; nearly 90% of disease notifications collected data on sex. Only disease notifications and periodic prevalence surveys collected data on sexual orientation (7.4% and 41.2%, respectively), and only periodic prevalence surveys collected data on gender identity (17.7%). All systems based on registries/vital records and multiple sources collected data on race/ethnicity. Most systems, regardless of type, collected only three of the following: sex, sexual orientation, gender identity, age, and race/ethnicity (100% of registries/vital records and multiple sources, 55.6% of disease notifications, and 52.9% of periodic prevalence surveys); in all cases, this included sex, age, and race/ethnicity. Periodic prevalence surveys and disease notifications were the only types of systems to collect more than three of these data types; 7.4% of disease notifications collected four, 23.5% and 17.7% of periodic prevalence surveys collected four and five, respectively (Table 3).

## 4. Discussion

Our study found variation in the degrees to which sex-related and additional demographic variables were collected and reported. All of the systems we assessed collected data on age, and most collected data on sex and race/ethnicity. Far fewer systems collected and reported data on sexual orientation and gender identity. Periodic prevalence surveys were among the most common system types for collecting all the variables we assessed; nearly 2 in 5 of these systems collected data on four or five demographic variable types. It is encouraging that most systems collected data on sex, age, and race/ethnicity; yet, the opportunity remains, particularly for certain data system types, to increase collection of data on SOGI variables.

Different diseases and conditions may affect men and women differently at different times in life; therefore, it is important to collect and report public health data by sex across the life course (e.g., before, during, and after reproductive years) to better understand how early physical or social life exposures influence health outcomes or disease risk across life. Recognizing this, government and other funding agencies are compelled to ensure participation of women in research and to require reporting of data by sex [1,4]. Encouragingly, the majority of U.S. public health data and monitoring systems we assessed collected data on sex. Periodic prevalence surveys, registries/vital records, and systems based on multiple sources all collected data on sex (as well as age), while disease notifications were less likely to do so. This may be due in part to language in the HHS standards that specifically mention population-based health surveys; applicability to other types of systems is not explicitly stated [37]. Some other possible reasons data systems may not measure a standard variable such as sex include perceived lack of relevance to the topic being measured, and potentially a desire to limit burden on respondents or need to limit resources required for maintaining a data system.

Few systems collected and reported data on sexual orientation and gender identity; those that did were most often periodic prevalence surveys or covered health topics historically considered highly related to sexual orientation and gender identity (e.g., HIV). In addition to the reasons described above, additional challenges exist when conducting health research into LGBT populations, including lack of consensus on definitions and measures of sexual orientation and gender identity, perceived (by researchers) or actual reluctance of study participants to disclose information about sexual orientation and gender identity, and, often, small sample sizes [15]. Additionally, SOGI is often a fluid construct that may be difficult to capture in cross-sectional studies; for example, a longitudinal study of LGB youth found that nearly one-fifth transitioned from bisexual to gay/lesbian over the course of one year [38,39]. A federal workgroup is working to address some of those challenges [40], and several federal data systems have successfully implemented SOGI measures [11]. Collecting data by sexual orientation and gender identity is an important step in identifying and addressing health disparities that disproportionately affect sexual minority groups.

All the systems we assessed collected data on age; approximately 80% collected data on race/ethnicity, which is similar to a previous review of CDC-sponsored data systems active between 2010 and 2013 [4]. We found that, overall, 3 out of every 5 systems collected data on the three variables age, sex, and race/ethnicity, while only 1 out of every 5 systems also collected data on sexual orientation and/or gender identity. Periodic prevalence surveys (followed by disease notifications) most commonly collected data on four or more of the demographic variable types we considered. Collecting data on demographic characteristics, such as those we considered, is important to allow better identification of health disparities (e.g., within demographic categories, such as female and male) and development of appropriate and effective public health interventions.

It is important not only to collect data on sex, sexual orientation, and gender identity but also to ensure that health-related data are analyzed and reported by sex-related variables, age, and race/ethnicity, among other important demographic variables not discussed here. CDC has increased efforts to improve surveillance systems through enhanced data collection, analysis, visualization, and dissemination. One example is by providing tools that allow for better access to, analysis, and visualization of data, such as data.cdc.gov [41]. It was beyond the scope of this study to determine whether, how, and at what frequency data collected by these systems were reported or presented by the demographic variable types we assessed. However, for these data to be most useful and informative, they need to be easy to find and understand, and ideally communication and dissemination plans would be developed to maximize their reach and utility.

There were a number of limitations to our study. First, this is not a comprehensive listing of U.S. public health data systems. We searched only certain websites and limited our assessment to CDC systems active from 2015–2018 that demonstrated ongoing data collection. Therefore, we excluded some online systems updated between these years that displayed only data from 2014 and before. We may also have erroneously excluded newer systems that collected data only once during that timeframe, as we were unable to determine the systems’ periodicity or ongoing collection. Second, it is possible we mis-categorized some data systems by type. Some data systems shared characteristics of multiple types, and some system owners may have considered their system to belong to one category, whereas we assigned it to another category based on our definitions. Third, we did not examine how data systems collected data on sex-related variables, age, or race/ethnicity. For example, some systems collected data only on broad racial (e.g., Black or African American, Asian) and ethnic (e.g., Hispanic) categories, while others collected data on detailed categories, such as Chinese or Cuban. Fourth, to our knowledge, no major data systems include items on intersex conditions; therefore, we were unable to assess data collection of this construct at this time. Finally, some data systems we identified did not provide publicly available data; thus, it was challenging for us to assess those systems. In these instances, we attempted to contact system owners; however, rarely was contact information provided. Despite these limitations, we were able to assess 51 data systems measuring a wide variety of health topics.

## 5. Conclusions

Availability of data disaggregated by sex, sexual orientation, gender identity, age, and race/ethnicity is an essential step toward eliminating gaps in knowledge that may be contributing to a continuation or widening of health disparities in these populations. While most U.S. public health data and monitoring systems collect data disaggregated by sex, age, and race/ethnicity, far fewer do so for sexual orientation or gender identity. For those systems that do not collect these vitally important data yet wish to do so, standards and examples exist to aid these efforts. Those that already collect these data may want to consider increasing data accessibility and disseminating appropriately tailored reports to public health professionals, policymakers, communities, and other collaborators.

## Figures and Tables

**Table 1:** Types of data systems, definitions, and examples ^1^.

Data System Type	Definition	Examples	Example System
Disease notification	Reporting of certain diseases or other health-related conditions by a specific group, as specified by law, regulation, or agreement	Notifiable disease case reports, notifications for adverse effects from medications or vaccines, sentinel reporting of conditions	Childhood Blood-Lead Poisoning Surveillance System (CBLS)
Periodic prevalence survey	A periodic or recurrent investigation designed to obtain specific information about a population of interest	HIV seroprevalence surveys, population-based health surveys	Behavioral Risk Factor Surveillance System (BRFSS)
Registry/vital record	Permanent record of persons or events	Disease registries (e.g., cancer or birth defects registries), immunization registries, vital event registries (e.g., birth and death records).	National Program of Cancer Registries (NPCR)
Multiple sources	A combination of the above-mentioned sources		National Quitline Data Warehouse (NQDW)

^1^ Based on categories identified from Principles of Epidemiology in Public Health Practice, 3rd Edition and Public Health Surveillance Toolkit: A Guide for Busy Task Managers [35,36].

**Table 2:** Selected summary characteristics of U.S.-based public health data and monitoring systems active between 2015–2018.

	All Systems (*n* = 51)	System Encompassed both Males and Females (*n* = 48)	Female-Specific System (*n* = 3)
	No. (%)	No. (%)	No. (%)
Collected data on sex-related variables			
Sex	48 (94.1%)	45 (93.8%)	3 (100%)
Sexual orientation	9 (17.7%)	9 (18.8%)	0 (0%)
Gender identity	3 (5.9%)	3 (6.3%)	0 (0%)
Collected data on additional demographic variables			
Age	51 (100%)	48 (100%)	3 (100%)
Race/ethnicity	40 (78.4%)	37 (77.1%)	3 (100%)
Type of system			
Disease notifications	27 (52.9%)	25 (52.1%)	2 (66.7%)
Periodic prevalence surveys	17 (33.3%)	16 (33.3%)	1 (33.3%)
Registries/vital records	6 (11.8%)	6 (12.5%)	0 (0%)
Multiple sources	1 (2.0%)	1 (2.1%)	0 (0%)

**Table 3:** Number and percentage of U.S. public health data and monitoring systems collecting sex-related variables, age, and race/ethnicity by type of data system, 2015–2018 ^1^.

	Disease Notifications (*n* = 27)	Periodic Prevalence Surveys (*n* = 17)	Registries/Vital Records (*n* = 6)	Multiple Sources (*n* = 1)
	No. (%)	No. (%)	No. (%)	No. (%)
Collected data on sex-related variables				
Sex	24 (88.9%)	17 (100%)	6 (100%)	1 (100%)
Sexual orientation	2 (7.4%)	7 (41.2%)	0 (0%)	0 (0%)
Gender identity	0 (0%)	3 (17.7%)	0 (0%)	0 (0%)
Collected data on additional demographic variables				
Age	27 (100%)	17 (100%)	6 (100%)	1 (100%)
Race/ethnicity	17 (63.0%)	16 (94.1%)	6 (100%)	1 (100%)
Number of demographic variable types collected ^2^				
1	3 (11.1%)	0 (0%)	0 (0%)	0 (0%)
2	7 (25.9%)	1 (5.9%)	0 (0%)	0 (0%)
3	15 (55.6%)	9 (52.9%)	6 (100%)	1 (100%)
4	2 (7.4)	4 (23.5%)	0 (0%)	0 (0%)
5	0 (0%)	3 (17.7%)	0 (0%)	0 (0%)

^1^ Includes 3 systems that capture data only on females. ^2^ 5 types assessed include (1) sex, (2) sexual orientation, (3) gender identity, (4) age, (5) race/ethnicity.

## Data Availability

No new data were created or analyzed in this study. Data sharing is not applicable to this article.
